# Middle lobe torsion following right upper lobectomy

**DOI:** 10.36416/1806-3756/e20240307

**Published:** 2024-12-17

**Authors:** Pedro Marx Nunes de Sousa, Felipe Welter Langer, Mariana Manica Tamiozzo

**Affiliations:** 1. Universidade Federal de Santa Maria, Santa Maria (RS) Brasil.; 2. Departamento de Radiologia e Diagnóstico por Imagem, Universidade Federal de Santa Maria, Santa Maria (RS) Brasil.

**Figure 1:**
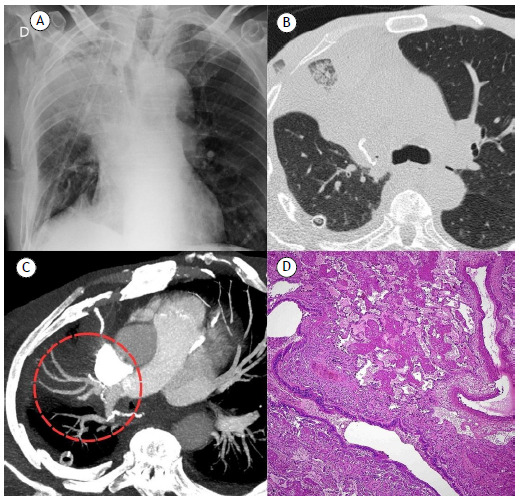
In A, anteroposterior chest X-ray showing atelectasis and cranial displacement of the middle lobe. In B and C, contrast-enhanced chest CT scans showing ground-glass opacities, interlobular septal thickening, and obstruction of the middle lobe bronchus, with no enhancement in the right middle lobe branches. In D, histopathological findings in the excised middle lobe, including areas of necrosis and activated pneumocytes, consistent with pulmonary parenchymal infarction.

A 69-year-old male patient was admitted for surgical excision of a right upper lobe adenocarcinoma measuring 8.1 cm × 7.2 cm × 5.5 cm. A right upper lobectomy with mediastinal lymphadenectomy was performed, and the adenocarcinoma was staged as pT4N0. On postoperative day 2, the patient presented with progressive dyspnea. An anteroposterior chest X-ray showed cranial displacement and atelectasis of the middle lobe (Figure 1A). A contrast-enhanced chest CT scan showed partial atelectasis of the middle lobe and obstruction of the middle lobe bronchus, as well as ground-glass opacities and smooth interlobular septal thickening (Figure 1B), with no enhancement in the middle lobe branches (Figure 1C). These findings were suggestive of middle lobe torsion. A middle lobectomy was performed, and histopathological examination confirmed the diagnosis of hemorrhagic infarction of the middle lobe (Figure 1D). 

Middle lobe torsion is a rare occurrence in which the middle lobe is twisted on its bronchovascular pedicle, often following right upper lobectomy and leading to hemorrhagic infarction. Symptoms are often nonspecific, including dyspnea, fever, and chest pain in the early postoperative period. Although chest CT may not always show evidence of torsion, indirect signs such as atelectasis, bronchial narrowing, and abnormal vascular enhancement can aid in the diagnosis. Management typically involves emergency lobectomy.^1^ ^-^ ^3^
